# miR-205-5p Mediated Downregulation of PTEN Contributes to Cisplatin Resistance in C13K Human Ovarian Cancer Cells

**DOI:** 10.3389/fgene.2018.00555

**Published:** 2018-11-19

**Authors:** Xiaoyan Shi, Lan Xiao, Xiaolu Mao, Jinrong He, Yu Ding, Jin Huang, Caixia Peng, Zihui Xu

**Affiliations:** ^1^Key Laboratory for Molecular Diagnosis of Hubei Province, The Central Hospital of Wuhan, Tongji Medical College, Huazhong University of Science and Technology, Wuhan, China; ^2^Department of Endocrinology & Metabolism, Renmin Hospital of Wuhan University, Wuhan, China; ^3^Central Laboratory, The Central Hospital of Wuhan, Tongji Medical College, Huazhong University of Science and Technology, Wuhan, China; ^4^Department of Obstetrics and Gynecology, The First Affiliated Hospital, An Hui Medical University, Hefei, China; ^5^Department of Clinical Laboratory, The Central Hospital of Wuhan, Tongji Medical College, Huazhong University of Science and Technology, Wuhan, China

**Keywords:** miR-205-5p, PTEN, cisplatin resistance, ovarian cancer, AKT

## Abstract

Cisplatin resistance is a major cause of treatment failure in advanced ovarian cancer. The limited evidence shows the paradoxical regulation of miR-205 on chemotherapy resistance in cancer. Herein, we found that miR-205-5p was enormously increased in cisplatin-resistant C13K ovarian cancer cells compared with its cisplatin-sensitive OV2008 parental cells using miRNA microarrays, which was further verified by quantitative PCR. Furthermore, we confirmed that inhibition of miR-205-5p upregulated PTEN and subsequently attenuated its downstream target p-AKT, which inversed C13K cells from cisplatin resistance to sensitivity. Our data suggest that miR-205-5p contributes to cisplatin resistance in C13K ovarian cancer cells may via targeting PTEN/AKT pathway.

## Introduction

Cisplatin is an anti-cancer chemotherapy drug which used to treat ovarian cancer in women who have already received surgery and/or radiation treatment. However, either resistance to cisplatin or multi-drug resistance (MDR) to cisplatin-centered chemotherapy is a major cause of treatment failure in human ovarian cancer. As we know, four well-characterized mechanisms have been suggested to account for cisplatin-resistance in cancer: (1) pre-target resistance – involving steps preceding the binding of cisplatin to DNA, (2) on-target resistance – directly related to DNA-cisplatin adducts, (3) post-target resistance – concerning the lethal signaling pathways elicited by cisplatin-mediated DNA damage, and (4) off-target resistance – affecting molecular circuitries that do not present obvious links with cisplatin-elicited signals (Galluzzi et al., 2012, 2014). Besides lots of genes involved, emerging evidences demonstrate that miRNAs such as miR-141 (Seki, 2011), miR-184 (Tung et al., 2016), miR-199a (Wang et al., 2013), miR-214 (Yang et al., 2008), and miR-421 (Ge et al., 2016) contribute to cisplatin-resistance in cancer. miR-205, a frequently silenced microRNA in cancer, has recently been implicated in chemotherapy resistance. De Cola et al. (2015) showed that upregulation of miR-205 leaded to lapatinib resistance in breast cancer stem cells via targeting ErbB/HER receptors. On the contrary, however, results from Puhr et al. (2012), Bhatnagar et al. (2010), and Alla et al. (2012) suggested that downregulation of miR-205 contributed to docetaxel or cisplatin resistance in prostate cancer, and cisplatin resistance in melanoma cells. Therefore, more extensive and detailed studies are required to explore the role of miR-205 in drug resistance associated with cancer chemotherapy.

In present study, we measured the miRNA expression profiles of cisplatin-resistant C13K ovarian cancer cell line compared with its cisplatin-sensitive OV2008 parent cell line using miRNA microarrays, and found that miR-205-5p was enormously increased in cisplatin-resistant C13K ovarian cancer cells, which was further verified by quantitative real-time PCR (qPCR). Furthermore, we investigated the effects of miR-205-5p on cisplatin resistance in ovarian cancer cells and the underlying mechanism, and confirmed that inhibition of miR-205-5p upregulated PTEN and subsequently attenuated its downstream target p-AKT, which inversed C13K cells from cisplatin resistance to sensitivity. Our data suggest that miR-205-5p contributes to cisplatin resistance in C13K ovarian cancer cells via targeting PTEN/AKT pathway.

## Materials and Methods

### Cell Culture and Transfection

The cisplatin-sensitive human ovarian cancer cell line OV2008 and its cisplatin-resistant clone C13K were supplied by Dr Wencheng Ding (Cancer Biology Research Center, Tongji Hospital, Tongji Medical College, Huazhong University of Science and Technology) (Asselin et al., 2001; Su et al., 2011). OV2008 cells were maintained in complete RPMI-1640 medium supplemented with 10% fetal bovine serum at 37°C in a humidified atmosphere containing 5% CO_2_. C13K cells were cultured in RPMI1640 supplemented with 10% fetal bovine-serum medium containing 1 μmol/L cisplatin to maintain resistance. The cells were transfected with 100 pmol hsa-miR-205-5p mimics or hsa-miR-205-5p inhibitor using Lipofectamine 2000 transfection reagent (Invitrogen, Carlsbad, CA, United States). The sequences for hsa-miR-205-5p are as follow: hsa-miR-205-5p mimics (5′-UCCUUCAUUCCACCGGAGUCUG-3′, 5′- GACUCCGGUGGAAUGAAGGAUU-3′); hsa-miR-205-5p mimics negative control (NC) (5′-UUCUCCGAACGUGUCACGUTT-3′, 5′-ACGUGACACGUUCGGAGAATT-3′); hsa-miR-205-5p inhibitor (5′-CAGACUCCGGUGGAAUGAAGGA-3′); mircoRNA inhibitor NC (5′-CAGUACUUUUGUGUAGUACAA -3′).

### Cytotoxicity Analysis

Cell cytotoxicity was analyzed with Cell Counting Kit-8 (CCK-8) assay kit (Sigma-Aldrich, St. Louis, MO, United States). Briefly, cells were seeded into flat-bottomed 96-well plates at a density of 8000 per well and incubated overnight. Cisplatin (Sigma-Aldrich, St. Louis, MO, United States) with concentrations of 0, 20, 40, 60, 80, 100 and 120 μM was added after adherence. Then cells were continuously cultured for 24 or 48 h followed by treatment with 10 ul of CCK-8 solution for additional 1h at 37°C, and the absorbance (A) was measured at 450 nm by an Enspire microplate reader (Perkin Elmer, United States). Cell viability (%) = experimental group A value/control group A value × 100. IC50 values (50% inhibition of surviving fraction) were then estimated using the fitted dose-response curves for cell viability.

### RNA Extraction and miRNA Microarray Analysis

Total RNA was extracted by Trizol reagent (Invitrogen, Carlsbad, CA, United States). The RNA sample was purified with an RNeasy Mini Column (Qiagen, CA, United States) and the RNA quality was assessed by 1.0% agarose gel electrophoresis. miRNA-profiling was carried out by miRNA microarray using Affymetrix miRNA 4.0 and Affymetrix GeneChip (Affymetrix, United States). The random-variance model (RVM) F test was applied to filter differentially expressed genes for the two ovarian cancer cell lines. After the significance analysis and false discovery rate (FDR) analysis, differentially expressed genes were selected according to their *p*-value threshold.

### qPCR

miRNAs expression was measured using TaqMan miRNA reverse transcription kit and TaqMan miRNA assay kits (Applied Biosystems, United States). Primer sequences for hsa-miR-205-5p: (5′-CCTTCATTCCACCGGAGT-3′; 5′-GTCCAGTTTTTTTTTTTTTTTCAGACT-3′). PTEN mRNA expression was measured using TaqMan mRNA reverse transcription kit and SYBR Green Supermix (BioRad, United States), primer sequences for PTEN: (5′-TGGATTCGACTTAGACTTGACCT; 5′-TTTGGCGGTGTCATA ATGTCTT).

### Cell Apoptosis Assay

Cell apoptosis was measured by Flow Cytometry. All samples were washed in phosphate-buffered saline and resuspended in 200 μl binding buffer. Next, 5 μl Annexin–V-fluorescein isothiocyanate and 10 μl propidium iodide (PI; 1 μg/ml) were added and the cell suspension was incubated in a dark chamber for 1h at room temperature. Cell apoptosis was then determined using a FACSCalibur flow cytometer (BD Biosciences, United States) and data were analyzed using CellQuest software (BD Biosciences, United States).

### Western Blotting Analysis

Cells were harvested with ice-cold PBS and lysed in lysis buffer containing a protease inhibitor cocktail. A total of 60 μg protein was separated by 10% SDS-PAGE and transferred to polyvinylidene fluoride membranes. Following blocking with Tris-buffered saline containing 5% skimmed milk for 1 h at room temperature, the membranes were incubated with the primary antibodies (anti-PTEN, anti-actin, anti-AKT, anti-phospho-AKT, Santa Cruz or Cell Signaling, United States) in blocking buffer overnight at 4°C. The membranes were washed three times in TBST and then incubated with horseradish peroxidase-conjugated anti-mouse/rabbit antibodies at a dilution of 1:3,000 for 1 h at room temperature. Signals were detected on X-ray film using an enhanced chemiluminescent detection system (Pierce Biotechnology, Inc., Rockford, IL, United States).

### Statistical Analysis

All experiments were repeated at least three times and the data were expressed as the mean ± standard deviation (SD). Statistical analysis was performed with SPSS 18.0 for Windows (SPSS, Inc., Chicago, IL, United States). Statistical significance was determined by the Student *t*-test or one-way ANOVA. *P* < 0.05 was considered to indicate a statistically significant difference.

## Results

### Differentially Expressed miRNAs in Cisplatin-Resistant Variant C13K Cells Versus Its Cisplatin-Sensitive OV2008 Parental Cells

**FIGURE 1:**
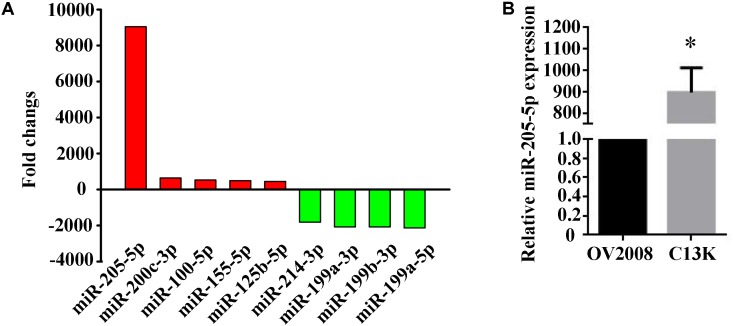
Elevated expression of miR-205-5p in cisplatin-resistant C13K cells compared with its cisplatin-sensitive OV2008 parental cells. **(A)** 9 representative differentially expressed miRNAs with statistically significant fold changes in C13K cells vs. OV2008 cells. Upregulated miRNAs (red bars) are shown above the x-axis, whereas downregulated miRNAs (green bars) below the x-axis. **(B)** qPCR validation of the differential expression of miR-205-5p in C13K cells vs. OV2008 cells. The data represent mean ± SD of three independent experiments (^∗^P < 0.01).

C13K cells are cisplatin-resistant variant originating from its cisplatin-sensitive OV2008 parental cells (Su et al., 2011). We used miRNA microarray to determine miRNA expression profiles in both C13K and OV2008 cell lines. We have identified 113 miRNAs that were significantly differentially expressed (GEO accession number: GSE120256) in C13K cells, including 32 upregulated miRNAs (such as hsa-miR-205-5p, hsa-miR-200c-3p, hsa-miR-100-5p, hsa-miR-155-5p, and hsa-miR-125b-5p) and 81 down-regulated miRNAs (such as hsa-miR-214-3p, hsa-miR-199a-3p, hsa-miR-199b-3p, hsa-miR-199a-5p), compared to OV2008 cells (Figure 1A). Surprisingly, miR-205-5p was around 9000 fold changes among 32 up-regulated miRNAs, which was further verified by qPCR (Figure 1B).

To investigate the role of miR-205, target prediction using softwares such as miRanda, TargetScan and PicTar was performed (data in Supplementary Data Sheets 1,2). Several of the predicted targets are known to be involved in cancer progression (E2F1, E2F5, ERBB, PTEN), invasion and metastasis (ZEB1/2, LRP-1). mirTarBase analysis was performed to further identify the experimentally validated targets of miR-205. These indicated that some but not all of these genes are aberrantly expressed in C13K cisplatin-resistant cells.

### miR-205-5p Attenuated Cisplatin-Induced Cytotoxicity

**FIGURE 2:**
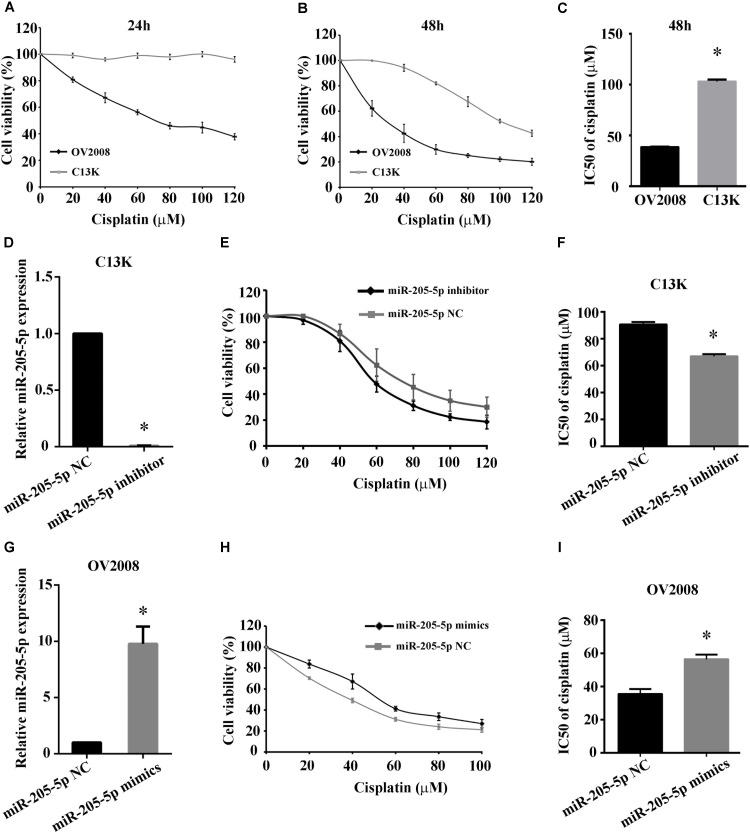
Overexpression of miR-205-5p reduced cisplatin-induced cytotoxicity in ovarian cancer cells. C13K and OV2008 cells were treated with cisplatin for 24 h **(A)** or 48 h **(B)** and then were assessed for cell viability by CCK-8 assay. IC50 values at 48 h were estimated using the fitted dose-response curves for cell viability **(C)**. Cells transfected with miR-205-5p inhibitor **(D–F)** or mimics **(G–I)** were treated with 40 μM cisplatin for 48 h. Cell viability was assessed by CCK-8 assay. miR-205-5p expression was measured by qPCR in C13K cells **(D–F)** or OV2008 cells **(G–I)**. The data represent mean ± SD of three independent experiments (^∗^*P* < 0.01).

We confirmed that the viability of C13K cells was significantly increased following treatment with cisplatin for 48 h compared to OV2008 cells, and the IC50 values of cisplatin in C13K cells (107 μmol/L) were higher than the corresponding values in OV2008 (37 μmol/L), which indicated that C13K cells exhibited cisplatin resistance (Figures 2A–C).

To investigate whether miR-205**-**5p is associated with cisplatin-induced cytotoxicity in ovarian cancer cells, we examined the cell viability of C13K and OV2008 cells transfected with miR-205**-**5p inhibitor or mimics following cisplatin treatment for 48 h. We observed that both cell viability and IC50 in C13K decreased upon downregulation of miR-205**-**5p (Figures 2D–F), whereas both of them in OV2008 elevated upon upregulation of miR-205**-**5p (Figures 2G–I). These data suggested that miR-205**-**5p attenuated cisplatin-induced cytotoxicity and enhanced cisplatin resistance in ovarian cancer cells.

### miR-205-5p Inhibits Cisplatin-Induced Apoptosis

**FIGURE 3:**
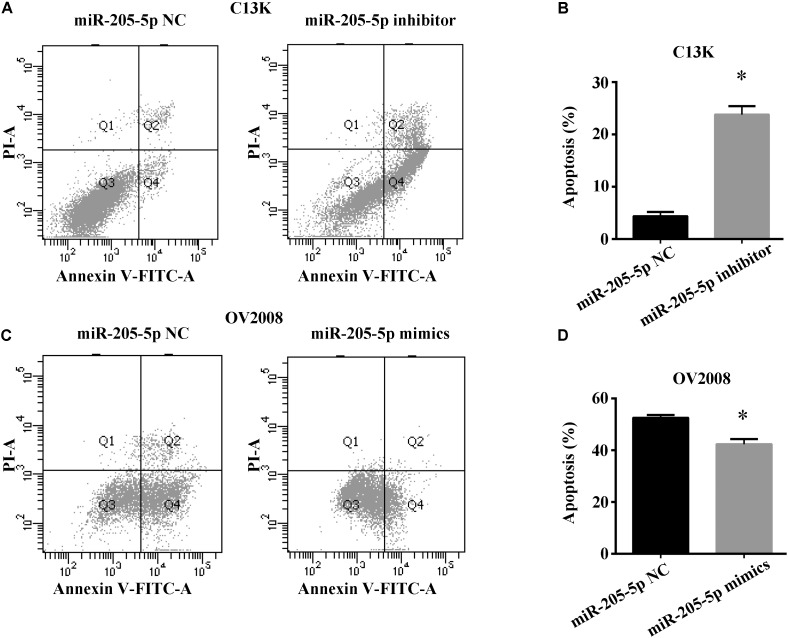
miR-205-5p inhibited cisplatin induced apoptosis in ovarian cancer cells. C13K **(A**,**B)** or OV2008 **(C,D)** cells were transfected with miR-205-5p inhibitor **(A,B)** or mimics **(C,D)**, and then were treated with 40 μM cisplatin for 48 h. Cell apoptosis were subjected to flow cytometry. Data represent the mean ± SD of three independent experiments (^∗^*P* < 0.01).

To explore whether miR-205**-**5p accounts for cisplatin-induced apoptosis in ovarian cancer cells, we measured cell apoptosis in both C13K and OV2008 cells transfected with miR-205**-**5p inhibitor or mimics following cisplatin treatment for 48 h. We found that C13K cells apoptosis increased upon downregulation of miR-205**-**5p (Figures 3A,B), whereas OV2008 cells apoptosis reduced upon upregulation of miR-205**-**5p (Figures 3C,D).

### miR-205-5p Contributes to Cisplatin-Resistance in Ovarian Cancer Cells via Targeting PTEN/AKT Pathway

**FIGURE 4:**
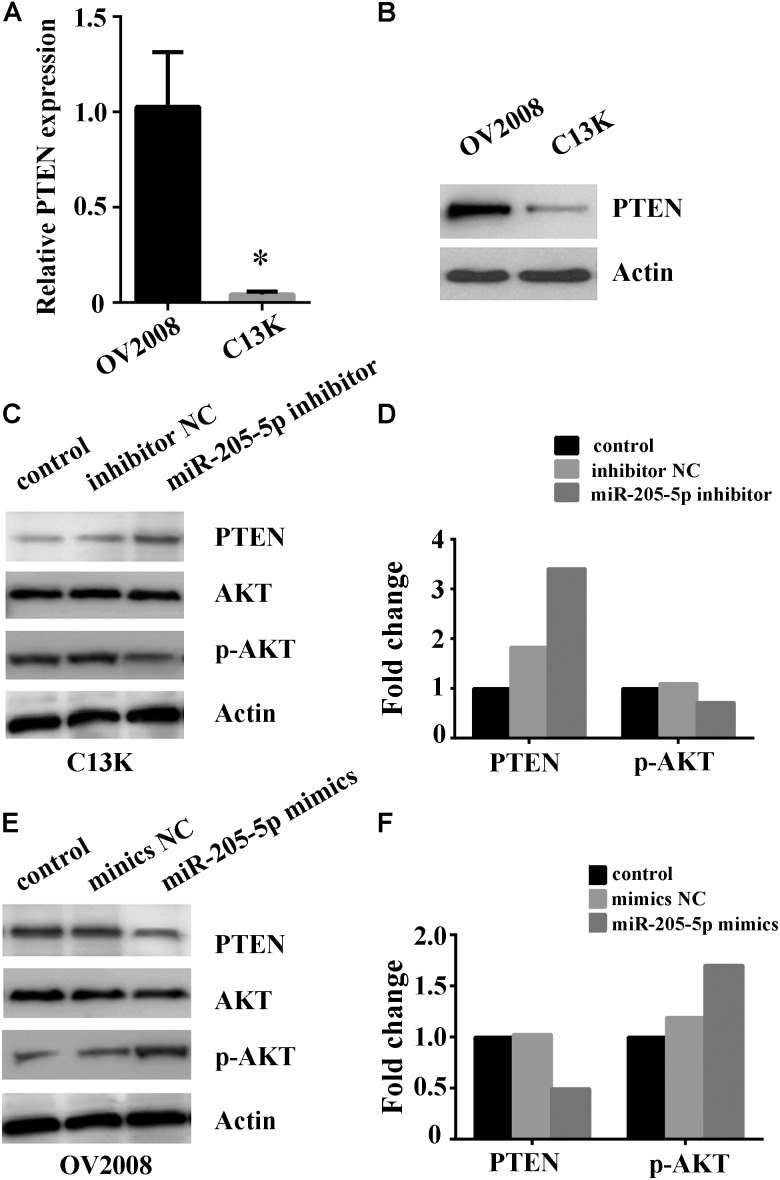
Involvement of miR-205-5p in cisplatin resistance via PTEN/AKT pathway in ovarian cancer cells. Relative expression of PTEN mRNA **(A)** and protein **(B)** in C13K and OV2008 cells (^∗^*P* < 0.01). **(C**,**D)** Inhibition of miR-205-5p increased PTEN and decreased p-AKT in C13K cells with cisplatin treatment for 48 h. **(E**,**F)** Overexpression of miR-205-5p decreased PTEN and increased p-AKT in OV2008 with cisplatin treatment for 48 h.

Given the involvement of PTEN in chemotherapy resistance including cisplatin in cancer (Lee et al., 2005; Juric et al., 2015), it is important to reveal whether PTEN, one of miR-205 target genes, is linked to miR-205 upregulation in cisplatin-resistant ovarian cancer cells. We examined PTEN mRNA and protein expression by using qPCR and Western blotting. Our results showed remarkable lower expression of PTEN mRNA and protein in C13K cells compared to that in OV2008 (Figures 4A,B). We further measured the expression of PTEN and AKT in C13K and OV2008 cells transfected with miR-205**-**5p inhibitor or mimics following cisplatin treatment for 48 h. We found that PTEN increased and phospho-AKT (p-AKT) decreased in C13K upon downregulation of miR-205**-**5p (Figures 4C,D). On the contrary, PTEN decreased and p-AKT increased in OV2008 upon upregulation of miR-205**-**5p (Figures 4E,F).

## Discussion

In the last few years, drug resistance in various cancers has been linked with aberrant expression of miRNAs, suggesting that miRNAs might play important roles in chemoresistance and chemosensitivity (Koster et al., 2013). Although resistance to current chemotherapeutics represents a significant barrier to the improvement of the long-term overcome of patients with ovarian cancer, chemotherapy is still one of the most effective therapies for ovarian cancer patients. Although altered miRNAs are differentially expressed in ovarian cancer, the potential role of miRNAs in the induction of drug resistance, particularly in platinum resistance, has not been fully investigated. Growing evidences demonstrate that more and more miRNAs contribute to cisplatin-resistance in cancer (Yang et al., 2008; Wang et al., 2013; Ge et al., 2016; Tung et al., 2016). In this study, we identified 113 miRNAs which were significantly differentially expressed, including 32 upregulated miRNAs and 81 down-regulated miRNAs. Most of them (such as miR-205, miR-200c, miR-100, miR-155, miR-125b, miR-214, miR-199a, miR-199b and miR-199a) are involved in cell differentiation, proliferation and apoptosis, and cancer development, progression and metastasis.

In present report, we suggests that upregulation of miR-205-5p contributes to cisplatin resistance in C13K ovarian cancer cells. However, evidences showed the paradoxical regulation of miR-205 on chemotherapy resistance in various cancers. Specifically, increased miR-205 level and paclitaxel resistance were noted in CD133+ ovarian cancer stem cells compared to adherent OVCAR3 cells (Nam et al., 2012). Additionally, increased miR-205 level was correlated with high proliferation, invasion and migration rates, enhanced resistance to carboplatin both in lung cancer A549 cells and H1975 cells by inhibiting cellular apoptosis and augmenting cancer cell survival (Zarogoulidis et al., 2015). However, downregulation of miR-205 was noted in pancreatic cancer cell lines resistant to gemcitabine (Bera et al., 2014) and prostate cancer cell lines resistant to docetaxel (Puhr et al., 2012). Overexpression of miR-205 resensitized drug-resistant cancer cells and acted as a tumor suppressor miRNA (Puhr et al., 2012; Mittal et al., 2014; Ippolito et al., 2016; Chaudhary et al., 2017). As we know, miR-205 is involved in both physiological and pathological processes including cell proliferation, apoptosis, angiogenesis and mesenchymal transition by targeting either oncogenes or tumor suppressor genes such as PHLPP-2, CTGF, CYR61, ESRRGr, PTEN, ErbB3, E2F1, E2F5, Zeb1&2, HBx, PCNA, Her 2&3, VEGF-A, Bcl2, etc (Vosgha et al., 2014), which results in “dual roles” of cell regulation. Nevertheless, more extensive and detailed studies are required to explore the dual role of miR-205 in drug resistance associated with cancer chemotherapy.

The tumor suppressor PTEN, one of miR-205 target genes, plays an essential role in cancer development via modulating cell cycle progression (Qu et al., 2012; Cai et al., 2013). It was found that PTEN deletion in malignant gliomas, endometrial cancer, melanomas, breast cancer and other tumors, indicating that the PTEN gene has an important function in suppressing transformation to a malignant tumor (Habib et al., 2011). PTEN levels were significantly decreased in progesterone-resistant endometrial cancer Ishikawa cells and that the miR-205 inhibitor increased PTEN expression in these cells (Zhuo and Yu, 2017). Besides, PTEN acts as a phosphatase to dephosphorylate phosphatidylinositol (3,4,5)-trisphosphate (PIP3), which results in inhibition of AKT signaling pathway (Cantley and Neel, 1999). It has been implicated that PTEN is involved in chemotherapy resistance including cisplatin in breast cancer, prostate cancer, melanoma, and EMT (Lee et al., 2005; Juric et al., 2015). Therefore, we speculated that miR-205 confers cisplatin resistance upon ovarian cancer cells through targeting PTEN. In this study, we confirmed that inhibition of miR-205-5p upregulated PTEN expression and subsequently attenuated its downstream target p-AKT, which inversed C13K cells from cisplatin resistance to sensitivity. Contrarily, overexpression of miR-205-5p downregulated PTEN expression and subsequently increased p-AKT level, which turned OV2008 cells from cisplatin sensitivity into resistance.

Our findings suggest that miR-205 plays an important role in the loss of PTEN expression and the development of cisplatin-resistant in ovarian cancer cells. Nevertheless, this research has limitation. All the data was based on cisplatin-resistant C13K ovarian cancer cells and its cisplatin-sensitive OV2008 parental cells. Therefore, we should note that differential expression patterns of miRNAs in C13K and OV2008 cells in this study are not applicable for ovarian cancer in general. More ovarian cancer cell lines need be checked by modulating miR-205 level and drug resistance. However, it was found that miR-205 regulated the proliferation and invasion of ovarian cancer OVCAR-3 cell line via suppressing PTEN/SMAD4 expression (Chu et al., 2018). miR-205 level was increased in ovarian cancer and it promoted the invasive behavior of ovarian cancer cell lines (OVCAR-5, OVCAR-8, and SKOV-3) (Wei et al., 2017). These studies support our conclusion, although the roles of miR-205 in the induction of drug resistance has not been investigated.

## Conclusion

Our data suggest that miR-205-5p mediates downregulation of PTEN and upregulation of p-AKT, which contributes to cisplatin-resistance in C13K human ovarian cancer cells. Biological or pharmacological intervention based on miR-205-5p may be a new promising strategy to inverse the cisplatin resistance in human ovarian cancer cell.

## Author Contributions

ZX and CP conceived and designed the experiments and explained the data. XS, LX, and XM performed the experiment. ZX, CP, XS, LX, and XM analyzed the content of the data with the help of JH, YD, and JH. XS, LX, and XM wrote the manuscript with the help of ZX and CP.

## Conflict of Interest Statement

The authors declare that the research was conducted in the absence of any commercial or financial relationships that could be construed as a potential conflict of interest. The reviewers SK, FW, and handling Editor declared their shared affiliation at the time of review.

## Supplementary Material

DATAT SHEET S2 miR-205 targets prediction using softwares such as miRanda, TargetScan, PicTar and mirTarBase analysis.

## References

[B1] Alla V. Kowtharapu B. S. Engelmann D. Emmrich S. Schmitz U. Steder M. (2012). E2F1 confers anticancer drug resistance by targeting ABC transporter family members and Bcl-2 via the p73/DNp73-miR-205 circuitry. *Cell Cycle* 11 3067–3078. 10.4161/cc.21476 22871739 PMC3442917

[B2] Asselin E. Mills G. B. Tsang B. K. (2001). XIAP regulates Akt activity and caspase-3-dependent cleavage during cisplatin-induced apoptosis in human ovarian epithelial cancer cells. *Cancer Res.* 61 1862–1868. 11280739

[B3] Bera A. VenkataSubbaRao K. Manoharan M. S. Hill P. Freeman J. W. (2014). A miRNA signature of chemoresistant mesenchymal phenotype identifies novel molecular targets associated with advanced pancreatic cancer. *PLoS One* 9:e106343. 10.1371/journal.pone.0106343 25184537 PMC4153643

[B4] Bhatnagar N. Li X. Padi S. K. Zhang Q. Tang M. S. Guo B. (2010). Downregulation of miR-205 and miR-31 confers resistance to chemotherapy-induced apoptosis in prostate cancer cells. *Cell Death Dis.* 1:e105. 10.1038/cddis.2010.85 21368878 PMC3004480

[B5] Cai J. Fang L. Huang Y. Li R. Yuan J. Yang Y. (2013). miR-205 targets PTEN and PHLPP2 to augment AKT signaling and drive malignant phenotypes in non-small cell lung cancer. *Cancer Res.* 73 5402–5415. 10.1158/0008-5472.can-13-0297 23856247

[B6] Cantley L. C. Neel B. G. (1999). New insights into tumor suppression: PTEN suppresses tumor formation by restraining the phosphoinositide 3-kinase/AKT pathway. *Proc. Natl. Acad. Sci. U.S.A.* 96 4240–4245. 10200246 10.1073/pnas.96.8.4240 PMC33561

[B7] Chaudhary A. K. Mondal G. Kumar V. Kattel K. Mahato R. I. (2017). Chemosensitization and inhibition of pancreatic cancer stem cell proliferation by overexpression of microRNA-205. *Cancer Lett.* 402 1–8. 10.1016/j.canlet.2017.05.007 28536008 PMC5673079

[B8] Chu P. Liang A. Jiang A. Zong L. (2018). miR-205 regulates the proliferation and invasion of ovarian cancer cells via suppressing PTEN/SMAD4 expression. *Oncol. Lett.* 15 7571–7578. 10.3892/ol.2018.8313 29725462 PMC5920363

[B9] De Cola A. Volpe S. Budani M. C. Ferracin M. Lattanzio R. Turdo A. (2015). miR-205-5p-mediated downregulation of ErbB/HER receptors in breast cancer stem cells results in targeted therapy resistance. *Cell Death Dis.* 6:e1823. 10.1038/cddis.2015.192 26181203 PMC4650737

[B10] Galluzzi L. Senovilla L. Vitale I. Michels J. Martins I. Kepp O. (2012). Molecular mechanisms of cisplatin resistance. *Oncogene* 31 1869–1883. 10.1038/onc.2011.384 21892204

[B11] Galluzzi L. Vitale I. Michels J. Brenner C. Szabadkai G. Harel-Bellan A. (2014). Systems biology of cisplatin resistance: past, present and future. *Cell Death Dis.* 5:e1257. 10.1038/cddis.2013.428 24874729 PMC4047912

[B12] Ge X. Liu X. Lin F. Li P. Liu K. Geng R. (2016). MicroRNA-421 regulated by HIF-1alpha promotes metastasis, inhibits apoptosis, and induces cisplatin resistance by targeting E-cadherin and caspase-3 in gastric cancer. *Oncotarget* 7 24466–24482. 10.18632/oncotarget.8228 27016414 PMC5029715

[B13] Habib S. L. Yadav A. Mahimainathan L. Valente A. J. (2011). Regulation of PI 3-K, PTEN, p53, and mTOR in malignant and benign tumors deficient in tuberin. *Genes Cancer* 2 1051–1060. 10.1177/1947601912445376 22737271 PMC3379569

[B14] Ippolito L. Marini A. Cavallini L. Morandi A. Pietrovito L. Pintus G. (2016). Metabolic shift toward oxidative phosphorylation in docetaxel resistant prostate cancer cells. *Oncotarget* 7 61890–61904. 10.18632/oncotarget.11301 27542265 PMC5308698

[B15] Juric D. Castel P. Griffith M. Griffith O. L. Won H. H. Ellis H. (2015). Convergent loss of PTEN leads to clinical resistance to a PI(3)Kalpha inhibitor. *Nature* 518 240–244. 10.1038/nature13948 25409150 PMC4326538

[B16] Koster R. van Vugt M. A. Timmer-Bosscha H. Gietema J. A. de Jong S. (2013). Unravelling mechanisms of cisplatin sensitivity and resistance in testicular cancer. *Expert Rev. Mol. Med.* 15:e12. 10.1017/erm.2013.13 24074238

[B17] Lee S. Choi E. J. Jin C. Kim D. H. (2005). Activation of PI3K/Akt pathway by PTEN reduction and PIK3CA mRNA amplification contributes to cisplatin resistance in an ovarian cancer cell line. *Gynecol. Oncol.* 97 26–34. 10.1016/j.ygyno.2004.11.051 15790433

[B18] Mittal A. Chitkara D. Behrman S. W. Mahato R. I. (2014). Efficacy of gemcitabine conjugated and miRNA-205 complexed micelles for treatment of advanced pancreatic cancer. *Biomaterials* 35 7077–7087. 10.1016/j.biomaterials.2014.04.053 24836307

[B19] Nam E. J. Lee M. Yim G. W. Kim J. H. Kim S. Kim S. W. (2012). MicroRNA profiling of a CD133(+) spheroid-forming subpopulation of the OVCAR3 human ovarian cancer cell line. *BMC Med. Genomics* 5:18. 10.1186/1755-8794-5-18 22643117 PMC3480901

[B20] Puhr M. Hoefer J. Schafer G. Erb H. H. Oh S. J. Klocker H. (2012). Epithelial-to-mesenchymal transition leads to docetaxel resistance in prostate cancer and is mediated by reduced expression of miR-200c and miR-205. *Am. J. Pathol.* 181 2188–2201. 10.1016/j.ajpath.2012.08.011 23041061

[B21] Qu C. Liang Z. Huang J. Zhao R. Su C. Wang S. (2012). MiR-205 determines the radioresistance of human nasopharyngeal carcinoma by directly targeting PTEN. *Cell Cycle* 11 785–796. 10.4161/cc.11.4.19228 22374676 PMC3356830

[B22] Seki N. (2011). A commentary on MicroRNA-141 confers resistance to cisplatin-induced apoptosis by targeting YAP1 in human esophageal squamous cell carcinoma. *J. Hum. Genet.* 56 339–340. 10.1038/jhg.2011.26 21390040

[B23] Su W. Huang L. Ao Q. Zhang Q. Tian X. Fang Y. (2011). Noscapine sensitizes chemoresistant ovarian cancer cells to cisplatin through inhibition of HIF-1alpha. *Cancer Lett.* 305 94–99. 10.1016/j.canlet.2011.02.031 21421285

[B24] Tung M. C. Lin P. L. Cheng Y. W. Wu D. W. Yeh S. D. Chen C. Y. (2016). Reduction of microRNA-184 by E6 oncoprotein confers cisplatin resistance in lung cancer via increasing Bcl-2. *Oncotarget* 7 32362–32374. 10.18632/oncotarget.8708 27083050 PMC5078019

[B25] Vosgha H. Salajegheh A. Smith R. A. Lam A. K. (2014). The important roles of miR-205 in normal physiology, cancers and as a potential therapeutic target. *Curr. Cancer Drug Targets* 14 621–637. 25308719 10.2174/156800961407140926105634

[B26] Wang Z. Ting Z. Li Y. Chen G. Lu Y. Hao X. (2013). microRNA-199a is able to reverse cisplatin resistance in human ovarian cancer cells through the inhibition of mammalian target of rapamycin. *Oncol. Lett.* 6 789–794. 10.3892/ol.2013.1448 24137412 PMC3789061

[B27] Wei J. Zhang L. Li J. Zhu S. Tai M. Mason C. W. (2017). MicroRNA-205 promotes cell invasion by repressing TCF21 in human ovarian cancer. *J. Ovarian Res.* 10:33. 10.1186/s13048-017-0328-1 28476165 PMC5420089

[B28] Yang H. Kong W. He L. Zhao J. J. O’Donnell J. D. Wang J. (2008). MicroRNA expression profiling in human ovarian cancer: miR-214 induces cell survival and cisplatin resistance by targeting PTEN. *Cancer Res.* 68 425–433. 10.1158/0008-5472.can-07-2488 18199536

[B29] Zarogoulidis P. Petanidis S. Kioseoglou E. Domvri K. Anestakis D. Zarogoulidis K. (2015). MiR-205 and miR-218 expression is associated with carboplatin chemoresistance and regulation of apoptosis via Mcl-1 and survivin in lung cancer cells. *Cell. Signal.* 27 1576–1588. 10.1016/j.cellsig.2015.04.009 25917317

[B30] Zhuo Z. Yu H. (2017). miR-205 inhibits cell growth by targeting AKT-mTOR signaling in progesterone-resistant endometrial cancer ishikawa cells. *Oncotarget* 8 28042–28051. 10.18632/oncotarget.15886 28427207 PMC5438629

