# Supplementary material for: miR-205-5p Mediated Downregulation of PTEN Contributes to Cisplatin Resistance in C13K Human Ovarian Cancer Cells
Source: Front Genet. 2018 Nov 19;9:555. doi: 10.3389/fgene.2018.00555 (PMC6253938; doi:10.3389/fgene.2018.00555)
Supplement: DATAT SHEET S2 — miR-205 targets prediction using softwares such as miRanda, TargetScan, PicTar and mirTarBase analysis. [file Data_Sheet_2.PDF]

miRNA	Rfam	Gene	miRanda	TargetScan	PicTar	mirTarBase
hsa-miR-205-5p	MI0000285	ABCD1	✔	✔		
hsa-miR-205-5p	MI0000285	ABI2	✔	✔		
hsa-miR-205-5p	MI0000285	ACACB	✔	✔		
hsa-miR-205-5p	MI0000285	ACSL1	✔	✔	✔	
hsa-miR-205-5p	MI0000285	ACSL4	✔	✔		
hsa-miR-205-5p	MI0000285	ADAMTS9	✔	✔	✔	
hsa-miR-205-5p	MI0000285	AEBP2	✔	✔	✔	
hsa-miR-205-5p	MI0000285	AFF1	✔	✔		
hsa-miR-205-5p	MI0000285	AGPAT6	✔	✔		
hsa-miR-205-5p	MI0000285	AKAP11		✔	✔	
hsa-miR-205-5p	MI0000285	AMOT	✔	✔	✔	
hsa-miR-205-5p	MI0000285	ANGPT2	✔	✔		
hsa-miR-205-5p	MI0000285	ANK2		✔	✔	
hsa-miR-205-5p	MI0000285	AP1AR	✔	✔		
hsa-miR-205-5p	MI0000285	AP1G1	✔	✔	✔	
hsa-miR-205-5p	MI0000285	ARFGEF1	✔		✔	
hsa-miR-205-5p	MI0000285	ATP7A	✔	✔		
hsa-miR-205-5p	MI0000285	AXIN2	✔	✔	✔	
hsa-miR-205-5p	MI0000285	B4GALT5	✔	✔		
hsa-miR-205-5p	MI0000285	B4GALT6	✔	✔		
hsa-miR-205-5p	MI0000285	BAMBI	✔	✔	✔	
hsa-miR-205-5p	MI0000285	BCL2	✔	✔		
hsa-miR-205-5p	MI0000285	BCL6	✔		✔	
hsa-miR-205-5p	MI0000285	BCL9L	✔	✔		
hsa-miR-205-5p	MI0000285	BMPER	✔	✔	✔	
hsa-miR-205-5p	MI0000285	BMPR1B	✔	✔		
hsa-miR-205-5p	MI0000285	BPTF	✔		✔	
hsa-miR-205-5p	MI0000285	BRCA1	✔	✔		
hsa-miR-205-5p	MI0000285	BTBD3	✔	✔	✔	
hsa-miR-205-5p	MI0000285	C10orf131	✔	✔		
hsa-miR-205-5p	MI0000285	C10orf53	✔	✔		
hsa-miR-205-5p	MI0000285	C11orf34	✔	✔		
hsa-miR-205-5p	MI0000285	C11orf86	✔	✔		
hsa-miR-205-5p	MI0000285	C12orf23	✔	✔		
hsa-miR-205-5p	MI0000285	C14orf101	✔	✔	✔	
hsa-miR-205-5p	MI0000285	C14orf43	✔	✔		
hsa-miR-205-5p	MI0000285	C14orf45	✔		✔	
hsa-miR-205-5p	MI0000285	C20orf111	✔	✔		
hsa-miR-205-5p	MI0000285	C21orf63	✔	✔		
hsa-miR-205-5p	MI0000285	C4orf34	✔		✔	
hsa-miR-205-5p	MI0000285	CADM1	✔	✔		
hsa-miR-205-5p	MI0000285	CALCRL	✔	✔	✔	
hsa-miR-205-5p	MI0000285	CALM1	✔	✔		
hsa-miR-205-5p	MI0000285	CALU	✔	✔	✔	
hsa-miR-205-5p	MI0000285	CANX	✔	✔	✔	
hsa-miR-205-5p	MI0000285	CAPZA1	✔		✔	
hsa-miR-205-5p	MI0000285	CASC4	✔	✔		
hsa-miR-205-5p	MI0000285	CASD1	✔	✔		
hsa-miR-205-5p	MI0000285	CBLL1	✔	✔		
hsa-miR-205-5p	MI0000285	CBX1	✔	✔		
hsa-miR-205-5p	MI0000285	CCDC43		✔	✔	
hsa-miR-205-5p	MI0000285	CCDC93	✔	✔	✔	
hsa-miR-205-5p	MI0000285	CCNJ	✔	✔	✔	
hsa-miR-205-5p	MI0000285	CDC27	✔	✔		

To be continued...

miRNA	Rfam	Gene	miRanda	TargetScan	PicTar	mirTarBase
hsa-miR-205-5p	MI0000285	CDC42BPB	✔	✔	✔	
hsa-miR-205-5p	MI0000285	CDH11	✔		✔	
hsa-miR-205-5p	MI0000285	CDK14	✔	✔		
hsa-miR-205-5p	MI0000285	CDK19	✔	✔	✔	
hsa-miR-205-5p	MI0000285	CDON	✔		✔	
hsa-miR-205-5p	MI0000285	CENPF	✔	✔		
hsa-miR-205-5p	MI0000285	CENPO	✔	✔		
hsa-miR-205-5p	MI0000285	CHD2	✔	✔		
hsa-miR-205-5p	MI0000285	CHIC1	✔	✔		
hsa-miR-205-5p	MI0000285	CHN1	✔	✔	✔	
hsa-miR-205-5p	MI0000285	CLDN11	✔	✔	✔	
hsa-miR-205-5p	MI0000285	CLDN8	✔	✔		
hsa-miR-205-5p	MI0000285	CLTC	✔	✔	✔	
hsa-miR-205-5p	MI0000285	CMTM4	✔	✔		
hsa-miR-205-5p	MI0000285	CNIH	✔		✔	
hsa-miR-205-5p	MI0000285	CNTLN	✔		✔	
hsa-miR-205-5p	MI0000285	COMMD10	✔	✔		
hsa-miR-205-5p	MI0000285	COX11	✔	✔		
hsa-miR-205-5p	MI0000285	CPEB2	✔	✔	✔	
hsa-miR-205-5p	MI0000285	CPSF6		✔	✔	
hsa-miR-205-5p	MI0000285	CSF1	✔	✔		
hsa-miR-205-5p	MI0000285	CSPP1	✔	✔		
hsa-miR-205-5p	MI0000285	CTGF				✔
hsa-miR-205-5p	MI0000285	CTPS2	✔	✔		
hsa-miR-205-5p	MI0000285	CUX2	✔	✔		
hsa-miR-205-5p	MI0000285	CXorf21	✔	✔		
hsa-miR-205-5p	MI0000285	CYR61				✔
hsa-miR-205-5p	MI0000285	DCHS1	✔	✔		
hsa-miR-205-5p	MI0000285	DDX5	✔	✔		✔
hsa-miR-205-5p	MI0000285	DDX52		✔	✔	
hsa-miR-205-5p	MI0000285	DDX6	✔	✔		
hsa-miR-205-5p	MI0000285	DGCR8	✔	✔	✔	
hsa-miR-205-5p	MI0000285	DHCR24	✔	✔		
hsa-miR-205-5p	MI0000285	DLG2	✔	✔	✔	
hsa-miR-205-5p	MI0000285	DMXL2	✔	✔		
hsa-miR-205-5p	MI0000285	DNAJA1	✔	✔		
hsa-miR-205-5p	MI0000285	DNM1L	✔	✔		
hsa-miR-205-5p	MI0000285	DOK4	✔	✔	✔	
hsa-miR-205-5p	MI0000285	DSC1	✔		✔	
hsa-miR-205-5p	MI0000285	DUSP7	✔	✔		
hsa-miR-205-5p	MI0000285	E2F1	✔	✔	✔	✔
hsa-miR-205-5p	MI0000285	E2F5	✔		✔	✔
hsa-miR-205-5p	MI0000285	EFCAB4A	✔		✔	
hsa-miR-205-5p	MI0000285	EFHA2	✔	✔		
hsa-miR-205-5p	MI0000285	EIF4E		✔	✔	
hsa-miR-205-5p	MI0000285	ELF1		✔	✔	
hsa-miR-205-5p	MI0000285	ENC1	✔	✔		
hsa-miR-205-5p	MI0000285	ENPP4	✔	✔		
hsa-miR-205-5p	MI0000285	EPB41	✔	✔		
hsa-miR-205-5p	MI0000285	EPB41L1	✔	✔		
hsa-miR-205-5p	MI0000285	EPS15	✔	✔		
hsa-miR-205-5p	MI0000285	ERBB2				✔
hsa-miR-205-5p	MI0000285	ERBB3	✔	✔	✔	✔
hsa-miR-205-5p	MI0000285	EREG	✔	✔	✔	

To be continued...

miRNA	Rfam	Gene	miRanda	TargetScan	PicTar	mirTarBase
hsa-miR-205-5p	MI0000285	ERRFI1	✔	✔		
hsa-miR-205-5p	MI0000285	ESM1	✔		✔	
hsa-miR-205-5p	MI0000285	ESRRG	✔	✔	✔	
hsa-miR-205-5p	MI0000285	ETF1	✔		✔	
hsa-miR-205-5p	MI0000285	ETNK1	✔	✔		
hsa-miR-205-5p	MI0000285	EZR	✔	✔		
hsa-miR-205-5p	MI0000285	FAM108B1	✔	✔		
hsa-miR-205-5p	MI0000285	FAM118B	✔	✔		
hsa-miR-205-5p	MI0000285	FAM120A	✔	✔	✔	
hsa-miR-205-5p	MI0000285	FAM120C	✔	✔		
hsa-miR-205-5p	MI0000285	FAM126A	✔	✔		
hsa-miR-205-5p	MI0000285	FAM155A	✔	✔		
hsa-miR-205-5p	MI0000285	FAM155B		✔	✔	
hsa-miR-205-5p	MI0000285	FAM176A	✔	✔		
hsa-miR-205-5p	MI0000285	FAM196A	✔	✔		
hsa-miR-205-5p	MI0000285	FAM84B	✔	✔	✔	
hsa-miR-205-5p	MI0000285	FBXO22	✔	✔	✔	
hsa-miR-205-5p	MI0000285	FERMT2	✔		✔	
hsa-miR-205-5p	MI0000285	FOXF1	✔	✔		
hsa-miR-205-5p	MI0000285	FRK	✔	✔	✔	
hsa-miR-205-5p	MI0000285	FZD3	✔	✔		
hsa-miR-205-5p	MI0000285	GAB1	✔	✔		
hsa-miR-205-5p	MI0000285	GATA3	✔	✔		
hsa-miR-205-5p	MI0000285	GCOM1	✔	✔		
hsa-miR-205-5p	MI0000285	GLIS3	✔	✔		
hsa-miR-205-5p	MI0000285	GPM6A	✔		✔	
hsa-miR-205-5p	MI0000285	GRAMD1C	✔	✔		
hsa-miR-205-5p	MI0000285	GRAMD2	✔	✔		
hsa-miR-205-5p	MI0000285	GTF3C2	✔	✔	✔	
hsa-miR-205-5p	MI0000285	GXYLT1	✔	✔		
hsa-miR-205-5p	MI0000285	H1F0	✔		✔	
hsa-miR-205-5p	MI0000285	HERC3	✔	✔		
hsa-miR-205-5p	MI0000285	HHLA1	✔	✔		
hsa-miR-205-5p	MI0000285	HIATL1	✔	✔		
hsa-miR-205-5p	MI0000285	HIF1AN	✔	✔		
hsa-miR-205-5p	MI0000285	HMG20A		✔	✔	
hsa-miR-205-5p	MI0000285	HMGB1	✔		✔	
hsa-miR-205-5p	MI0000285	HNRNPH3	✔	✔	✔	
hsa-miR-205-5p	MI0000285	HNRNPK	✔	✔	✔	
hsa-miR-205-5p	MI0000285	HS3ST1	✔	✔	✔	
hsa-miR-205-5p	MI0000285	HS3ST4	✔	✔		
hsa-miR-205-5p	MI0000285	HSD17B11	✔	✔	✔	
hsa-miR-205-5p	MI0000285	HSF5	✔	✔		
hsa-miR-205-5p	MI0000285	IFI44L	✔	✔		
hsa-miR-205-5p	MI0000285	IKZF4	✔	✔		
hsa-miR-205-5p	MI0000285	IL1R1	✔	✔		
hsa-miR-205-5p	MI0000285	IL24				✔
hsa-miR-205-5p	MI0000285	IL32				✔
hsa-miR-205-5p	MI0000285	INHBA	✔	✔	✔	
hsa-miR-205-5p	MI0000285	INPP4A	✔		✔	
hsa-miR-205-5p	MI0000285	INPPL1	✔	✔	✔	✔
hsa-miR-205-5p	MI0000285	IPO7		✔	✔	
hsa-miR-205-5p	MI0000285	IVNS1ABP	✔	✔	✔	
hsa-miR-205-5p	MI0000285	JPH4	✔	✔	✔	

To be continued...

miRNA	Rfam	Gene	miRanda	TargetScan	PicTar	mirTarBase
hsa-miR-205-5p	MI0000285	KCND2	✔		✔	
hsa-miR-205-5p	MI0000285	KIAA1429	✔	✔		
hsa-miR-205-5p	MI0000285	KIF1B	✔		✔	
hsa-miR-205-5p	MI0000285	KIF26B	✔	✔		
hsa-miR-205-5p	MI0000285	KLF12		✔	✔	
hsa-miR-205-5p	MI0000285	KLHL15	✔	✔		
hsa-miR-205-5p	MI0000285	KLHL30	✔		✔	
hsa-miR-205-5p	MI0000285	KPNA1	✔	✔		
hsa-miR-205-5p	MI0000285	KY	✔	✔		
hsa-miR-205-5p	MI0000285	LAMC1	✔	✔	✔	✔
hsa-miR-205-5p	MI0000285	LCA5	✔	✔		
hsa-miR-205-5p	MI0000285	LCOR	✔	✔	✔	
hsa-miR-205-5p	MI0000285	LHFPL2	✔	✔	✔	
hsa-miR-205-5p	MI0000285	LIMS2	✔	✔		
hsa-miR-205-5p	MI0000285	LIN9	✔	✔	✔	
hsa-miR-205-5p	MI0000285	LMNA	✔		✔	
hsa-miR-205-5p	MI0000285	LPAR1	✔	✔		
hsa-miR-205-5p	MI0000285	LPCAT1	✔	✔		
hsa-miR-205-5p	MI0000285	LRP1	✔	✔	✔	✔
hsa-miR-205-5p	MI0000285	LRP4	✔	✔		
hsa-miR-205-5p	MI0000285	LRP6	✔	✔		
hsa-miR-205-5p	MI0000285	LRPPRC	✔	✔		
hsa-miR-205-5p	MI0000285	LRRC58	✔	✔		
hsa-miR-205-5p	MI0000285	LRRK2	✔	✔		✔
hsa-miR-205-5p	MI0000285	LUC7L3	✔	✔	✔	
hsa-miR-205-5p	MI0000285	LY75	✔	✔		
hsa-miR-205-5p	MI0000285	LYPD6	✔	✔		
hsa-miR-205-5p	MI0000285	LYSMD3	✔	✔		
hsa-miR-205-5p	MI0000285	MAGI1	✔	✔		
hsa-miR-205-5p	MI0000285	MAGI2	✔	✔		
hsa-miR-205-5p	MI0000285	MAP3K13	✔	✔		
hsa-miR-205-5p	MI0000285	MARCKS	✔	✔	✔	
hsa-miR-205-5p	MI0000285	MED1	✔	✔	✔	✔
hsa-miR-205-5p	MI0000285	MFNG	✔	✔		
hsa-miR-205-5p	MI0000285	MGA	✔	✔		
hsa-miR-205-5p	MI0000285	MGAT4A	✔	✔		
hsa-miR-205-5p	MI0000285	MGRN1	✔	✔	✔	
hsa-miR-205-5p	MI0000285	MID1IP1	✔	✔	✔	
hsa-miR-205-5p	MI0000285	MKNK1	✔		✔	
hsa-miR-205-5p	MI0000285	MMAB	✔	✔		
hsa-miR-205-5p	MI0000285	MMD	✔	✔	✔	
hsa-miR-205-5p	MI0000285	MORF4L2	✔	✔		
hsa-miR-205-5p	MI0000285	MSL2	✔		✔	
hsa-miR-205-5p	MI0000285	MYO5B	✔	✔		
hsa-miR-205-5p	MI0000285	NAA11	✔	✔		
hsa-miR-205-5p	MI0000285	NAA25	✔	✔	✔	
hsa-miR-205-5p	MI0000285	NACC2	✔	✔		
hsa-miR-205-5p	MI0000285	NCOA1	✔	✔		
hsa-miR-205-5p	MI0000285	NDUFA4	✔	✔	✔	
hsa-miR-205-5p	MI0000285	NECAP1	✔		✔	
hsa-miR-205-5p	MI0000285	NEK6	✔	✔		
hsa-miR-205-5p	MI0000285	NEU1	✔	✔		
hsa-miR-205-5p	MI0000285	NFAT5		✔	✔	
hsa-miR-205-5p	MI0000285	NFIB	✔	✔		

To be continued...

miRNA	Rfam	Gene	miRanda	TargetScan	PicTar	mirTarBase
hsa-miR-205-5p	MI0000285	NHS	✔		✔	
hsa-miR-205-5p	MI0000285	NKD1	✔	✔		
hsa-miR-205-5p	MI0000285	NKX2-3	✔	✔		
hsa-miR-205-5p	MI0000285	NOTCH2	✔	✔		
hsa-miR-205-5p	MI0000285	NR3C2	✔	✔		
hsa-miR-205-5p	MI0000285	NSF	✔	✔		
hsa-miR-205-5p	MI0000285	OCIAD1	✔	✔		
hsa-miR-205-5p	MI0000285	PAFAH1B1	✔		✔	
hsa-miR-205-5p	MI0000285	PAPD5	✔	✔		
hsa-miR-205-5p	MI0000285	PAPLN	✔	✔	✔	
hsa-miR-205-5p	MI0000285	PAX9	✔	✔		
hsa-miR-205-5p	MI0000285	PCDH20	✔	✔		
hsa-miR-205-5p	MI0000285	PCNX	✔	✔		
hsa-miR-205-5p	MI0000285	PDE3B	✔	✔	✔	
hsa-miR-205-5p	MI0000285	PDS5A	✔	✔		
hsa-miR-205-5p	MI0000285	PEG3	✔	✔		
hsa-miR-205-5p	MI0000285	PHB	✔		✔	
hsa-miR-205-5p	MI0000285	PHC2	✔	✔	✔	
hsa-miR-205-5p	MI0000285	PHF16	✔	✔		
hsa-miR-205-5p	MI0000285	PHF17	✔	✔		
hsa-miR-205-5p	MI0000285	PHYHIPL	✔	✔		
hsa-miR-205-5p	MI0000285	PI16	✔	✔		
hsa-miR-205-5p	MI0000285	PICALM	✔	✔		
hsa-miR-205-5p	MI0000285	PJA2	✔	✔	✔	
hsa-miR-205-5p	MI0000285	PLCB1	✔	✔	✔	
hsa-miR-205-5p	MI0000285	PLEK	✔	✔		
hsa-miR-205-5p	MI0000285	PPP1R15B	✔	✔	✔	
hsa-miR-205-5p	MI0000285	PPP1R3A	✔		✔	
hsa-miR-205-5p	MI0000285	PPP1R8	✔	✔		
hsa-miR-205-5p	MI0000285	PPP4R4	✔		✔	
hsa-miR-205-5p	MI0000285	PRKCE	✔	✔		✔
hsa-miR-205-5p	MI0000285	PTCHD1	✔	✔		
hsa-miR-205-5p	MI0000285	PTEN	✔	✔		
hsa-miR-205-5p	MI0000285	PTK7	✔	✔		
hsa-miR-205-5p	MI0000285	PTP4A1	✔	✔		
hsa-miR-205-5p	MI0000285	PTP4A2	✔	✔		
hsa-miR-205-5p	MI0000285	PTPRJ	✔	✔		
hsa-miR-205-5p	MI0000285	PTPRM	✔	✔		
hsa-miR-205-5p	MI0000285	PUM1	✔		✔	
hsa-miR-205-5p	MI0000285	QKI	✔		✔	
hsa-miR-205-5p	MI0000285	RAB11FIP1	✔	✔	✔	
hsa-miR-205-5p	MI0000285	RAB14	✔	✔		
hsa-miR-205-5p	MI0000285	RAD17	✔	✔	✔	
hsa-miR-205-5p	MI0000285	RAP2B	✔	✔		
hsa-miR-205-5p	MI0000285	RARA	✔	✔		
hsa-miR-205-5p	MI0000285	RBM12	✔	✔		
hsa-miR-205-5p	MI0000285	RBM47	✔	✔	✔	
hsa-miR-205-5p	MI0000285	RBMS1	✔	✔		
hsa-miR-205-5p	MI0000285	RBPMS2	✔	✔		
hsa-miR-205-5p	MI0000285	RGS6	✔	✔		
hsa-miR-205-5p	MI0000285	RND3	✔		✔	
hsa-miR-205-5p	MI0000285	RNF157	✔	✔		
hsa-miR-205-5p	MI0000285	RNF213	✔	✔		
hsa-miR-205-5p	MI0000285	RNF4		✔	✔	

To be continued...

miRNA	Rfam	Gene	miRanda	TargetScan	PicTar	mirTarBase
hsa-miR-205-5p	MI0000285	RORA	✔	✔		
hsa-miR-205-5p	MI0000285	RPS6KA3	✔	✔		
hsa-miR-205-5p	MI0000285	RTN3	✔		✔	
hsa-miR-205-5p	MI0000285	RUNX2	✔	✔		
hsa-miR-205-5p	MI0000285	SATB2	✔	✔	✔	
hsa-miR-205-5p	MI0000285	SBF2	✔	✔	✔	
hsa-miR-205-5p	MI0000285	SCD5	✔	✔		
hsa-miR-205-5p	MI0000285	SCMH1	✔	✔	✔	
hsa-miR-205-5p	MI0000285	SEH1L	✔		✔	
hsa-miR-205-5p	MI0000285	SELT	✔	✔		
hsa-miR-205-5p	MI0000285	SEMA4C	✔	✔	✔	
hsa-miR-205-5p	MI0000285	SEMA7A	✔	✔		
hsa-miR-205-5p	MI0000285	SEPT11	✔	✔	✔	
hsa-miR-205-5p	MI0000285	SEPT4	✔		✔	
hsa-miR-205-5p	MI0000285	SERTAD2	✔	✔		
hsa-miR-205-5p	MI0000285	SGMS1	✔		✔	
hsa-miR-205-5p	MI0000285	SH2D4A	✔	✔		
hsa-miR-205-5p	MI0000285	SH3BGRL3	✔	✔		
hsa-miR-205-5p	MI0000285	SHROOM3	✔	✔		
hsa-miR-205-5p	MI0000285	SIAH1	✔	✔	✔	
hsa-miR-205-5p	MI0000285	SIGMAR1				✔
hsa-miR-205-5p	MI0000285	SLC19A2	✔	✔		
hsa-miR-205-5p	MI0000285	SLC30A8	✔	✔		
hsa-miR-205-5p	MI0000285	SLC35A1	✔	✔		
hsa-miR-205-5p	MI0000285	SLC35B3	✔	✔		
hsa-miR-205-5p	MI0000285	SLC4A4	✔	✔		
hsa-miR-205-5p	MI0000285	SLFN5	✔		✔	
hsa-miR-205-5p	MI0000285	SMAD1		✔	✔	
hsa-miR-205-5p	MI0000285	SMAD4	✔	✔		
hsa-miR-205-5p	MI0000285	SMG7	✔		✔	
hsa-miR-205-5p	MI0000285	SNX27		✔	✔	
hsa-miR-205-5p	MI0000285	SORBS1	✔	✔	✔	
hsa-miR-205-5p	MI0000285	SP4	✔	✔		
hsa-miR-205-5p	MI0000285	SP6	✔	✔		
hsa-miR-205-5p	MI0000285	SPATA13	✔	✔		
hsa-miR-205-5p	MI0000285	SPOPL	✔	✔		
hsa-miR-205-5p	MI0000285	SRC				✔
hsa-miR-205-5p	MI0000285	SRGAP1	✔	✔		
hsa-miR-205-5p	MI0000285	SS18	✔	✔		
hsa-miR-205-5p	MI0000285	STK3	✔	✔	✔	
hsa-miR-205-5p	MI0000285	STRBP	✔	✔	✔	
hsa-miR-205-5p	MI0000285	STS	✔	✔		
hsa-miR-205-5p	MI0000285	SULF1	✔	✔		
hsa-miR-205-5p	MI0000285	SUSD1	✔	✔		
hsa-miR-205-5p	MI0000285	SYPL2	✔	✔		
hsa-miR-205-5p	MI0000285	SYT13	✔	✔		
hsa-miR-205-5p	MI0000285	SYT9	✔	✔		
hsa-miR-205-5p	MI0000285	TAPT1	✔	✔		
hsa-miR-205-5p	MI0000285	TBX18	✔	✔		
hsa-miR-205-5p	MI0000285	TBX3	✔	✔		
hsa-miR-205-5p	MI0000285	TC2N	✔	✔		
hsa-miR-205-5p	MI0000285	TET1	✔	✔		
hsa-miR-205-5p	MI0000285	TFE3	✔	✔		
hsa-miR-205-5p	MI0000285	TGFA	✔	✔		

To be continued...

miRNA	Rfam	Gene	miRanda	TargetScan	PicTar	mirTarBase
hsa-miR-205-5p	MI0000285	TIAL1	✔	✔		
hsa-miR-205-5p	MI0000285	TIMM17A	✔	✔		
hsa-miR-205-5p	MI0000285	TLK1	✔	✔		
hsa-miR-205-5p	MI0000285	TM9SF3	✔	✔	✔	
hsa-miR-205-5p	MI0000285	TMEM136	✔	✔		
hsa-miR-205-5p	MI0000285	TMEM26	✔	✔		
hsa-miR-205-5p	MI0000285	TNFAIP8	✔	✔		
hsa-miR-205-5p	MI0000285	TNPO1	✔	✔		
hsa-miR-205-5p	MI0000285	TP53BP2	✔	✔	✔	
hsa-miR-205-5p	MI0000285	TP53INP1	✔	✔		
hsa-miR-205-5p	MI0000285	TP73				✔
hsa-miR-205-5p	MI0000285	TPP2	✔	✔		
hsa-miR-205-5p	MI0000285	TRAK2	✔	✔		
hsa-miR-205-5p	MI0000285	TRPS1	✔	✔	✔	
hsa-miR-205-5p	MI0000285	TSC22D1	✔		✔	
hsa-miR-205-5p	MI0000285	TSHZ3	✔	✔		
hsa-miR-205-5p	MI0000285	TTC19	✔	✔		
hsa-miR-205-5p	MI0000285	TTI1		✔	✔	
hsa-miR-205-5p	MI0000285	TXNRD1	✔	✔	✔	
hsa-miR-205-5p	MI0000285	UBE2E3	✔		✔	
hsa-miR-205-5p	MI0000285	UBE2G1	✔	✔	✔	
hsa-miR-205-5p	MI0000285	UBE2N	✔	✔	✔	
hsa-miR-205-5p	MI0000285	UBFD1		✔	✔	
hsa-miR-205-5p	MI0000285	UBIAD1	✔	✔		
hsa-miR-205-5p	MI0000285	UBQLN2	✔		✔	
hsa-miR-205-5p	MI0000285	UNC5C	✔	✔		
hsa-miR-205-5p	MI0000285	USP13	✔	✔		
hsa-miR-205-5p	MI0000285	USP48	✔		✔	
hsa-miR-205-5p	MI0000285	VASN	✔	✔	✔	
hsa-miR-205-5p	MI0000285	VEGFA	✔	✔		✔
hsa-miR-205-5p	MI0000285	VIP	✔	✔		
hsa-miR-205-5p	MI0000285	VTI1B	✔		✔	
hsa-miR-205-5p	MI0000285	WDR35	✔	✔		
hsa-miR-205-5p	MI0000285	WDR77	✔		✔	
hsa-miR-205-5p	MI0000285	WDTC1		✔	✔	
hsa-miR-205-5p	MI0000285	WHSC1	✔	✔	✔	
hsa-miR-205-5p	MI0000285	WHSC1L1	✔	✔		
hsa-miR-205-5p	MI0000285	WWC1	✔	✔		
hsa-miR-205-5p	MI0000285	WWC2	✔	✔		
hsa-miR-205-5p	MI0000285	WWC3	✔	✔		
hsa-miR-205-5p	MI0000285	WWP2	✔	✔		
hsa-miR-205-5p	MI0000285	XPO4	✔	✔		
hsa-miR-205-5p	MI0000285	YES1	✔	✔	✔	✔
hsa-miR-205-5p	MI0000285	ZCCHC14	✔	✔		
hsa-miR-205-5p	MI0000285	ZEB1	✔	✔		✔
hsa-miR-205-5p	MI0000285	ZEB2	✔		✔	✔
hsa-miR-205-5p	MI0000285	ZFYVE16	✔	✔		
hsa-miR-205-5p	MI0000285	ZHX3		✔	✔	
hsa-miR-205-5p	MI0000285	ZNF436	✔	✔		
hsa-miR-205-5p	MI0000285	ZNF536	✔	✔		
hsa-miR-205-5p	MI0000285	ZNF606	✔	✔		

(END)
